# A Combined Radiomics and Machine Learning Approach to Distinguish Clinically Significant Prostate Lesions on a Publicly Available MRI Dataset

**DOI:** 10.3390/jimaging7100215

**Published:** 2021-10-18

**Authors:** Leandro Donisi, Giuseppe Cesarelli, Anna Castaldo, Davide Raffaele De Lucia, Francesca Nessuno, Gaia Spadarella, Carlo Ricciardi

**Affiliations:** 1 Department of Advanced Biomedical Sciences, University of Naples “Federico II”, 80131 Naples, Italy; leandro.donisi@unina.it (L.D.); annacastaldo1202@gmail.com (A.C.); dav.delucia@gmail.com (D.R.D.L.); francesca.nessuno@gmail.com (F.N.); gaia.spadarella@gmail.com (G.S.); 2 Bioengineering Unit, Institute of Care and Scientific Research Maugeri, 27100 Pavia, Italy; giuseppe.cesarelli@unina.it; 3 Department of Chemical, Materials and Production Engineering, University of Naples “Federico II”, 80125 Naples, Italy; 4 Department of Electrical Engineering and Information Technologies, University of Naples “Federico II”, 80125 Naples, Italy

**Keywords:** radiomics, machine learning, MRI, prostate cancer

## Abstract

Although prostate cancer is one of the most common causes of mortality and morbidity in advancing-age males, early diagnosis improves prognosis and modifies the therapy of choice. The aim of this study was the evaluation of a combined radiomics and machine learning approach on a publicly available dataset in order to distinguish a clinically significant from a clinically non-significant prostate lesion. A total of 299 prostate lesions were included in the analysis. A univariate statistical analysis was performed to prove the goodness of the 60 extracted radiomic features in distinguishing prostate lesions. Then, a 10-fold cross-validation was used to train and test some models and the evaluation metrics were calculated; finally, a hold-out was performed and a wrapper feature selection was applied. The employed algorithms were Naïve bayes, K nearest neighbour and some tree-based ones. The tree-based algorithms achieved the highest evaluation metrics, with accuracies over 80%, and area-under-the-curve receiver-operating characteristics below 0.80. Combined machine learning algorithms and radiomics based on clinical, routine, multiparametric, magnetic-resonance imaging were demonstrated to be a useful tool in prostate cancer stratification.

## 1. Introduction

According to America Cancer Society, the estimated numbers of new cases and deaths from prostate cancer in the USA in 2021 are more than 240,000 and over 30,000, respectively [1]. As the prognosis of prostate cancer is strictly related to its biologically aggressive behavior, early detection and accurate risk stratification play a key role in ensuring the best outcome for patients [2]. In summary, clinically significant prostate cancer needs to be discriminated from low-grade disease to propose an adequate treatment to the patient [3]. To this end, magnetic resonance imaging (MRI) emerged as the most accurate imaging modality for the detection of clinically significant prostate cancer and actually plays a major role in the diagnostic pathway of the disease, since MRI is able to guide targeted biopsies [4,5]. Nevertheless, this technique has some limitations, such as the contrast-agent administration, a moderate specificity and the need for a high level of expertise to be correctly interpreted [6,7].

In recent years, radiomics and machine learning (ML) have shown their potential to extract quantitative features and elaborate them with complex algorithms to improve both the diagnosis and prognosis of patients.

Several authors demonstrated the advantage of the application of radiomics and ML, not only in prostate cancer but also in other fields of oncology [8,9,10]. In addition, recently, systematic reviews described the promising role of these techniques in prostate cancer [11,12,13,14,15]. The results of these studies suggested that, while MRI radiomics and ML approaches can reach high diagnostic accuracy in detecting severe prostate cancer and thus should be further investigated, the high heterogeneity of these studies has prevented their application in real life, indicating the need for standardized pipelines and the concomitant use of reliable benchmarks.

As a result, the aim of the present study is to evaluate the ability of the combined radiomics and ML approach using several ML algorithms (tree-based, instance-based and based on the a priori probability theory) on a publicly available dataset of MRI images, elaborated by Cuocolo et al. [16], in differentiating a clinically significant from a clinically non-significant prostate lesion.

**Figure 1:**
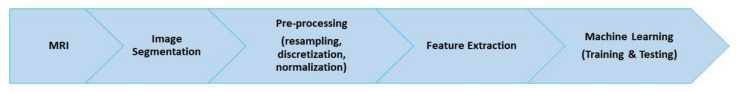
Workflow of the research.

Figure 1 summarizes the research workflow, which starts with MRI acquisition and ends with ML analysis.

## 2. Materials and Methods

### 2.1. Dataset

**Figure 2:**
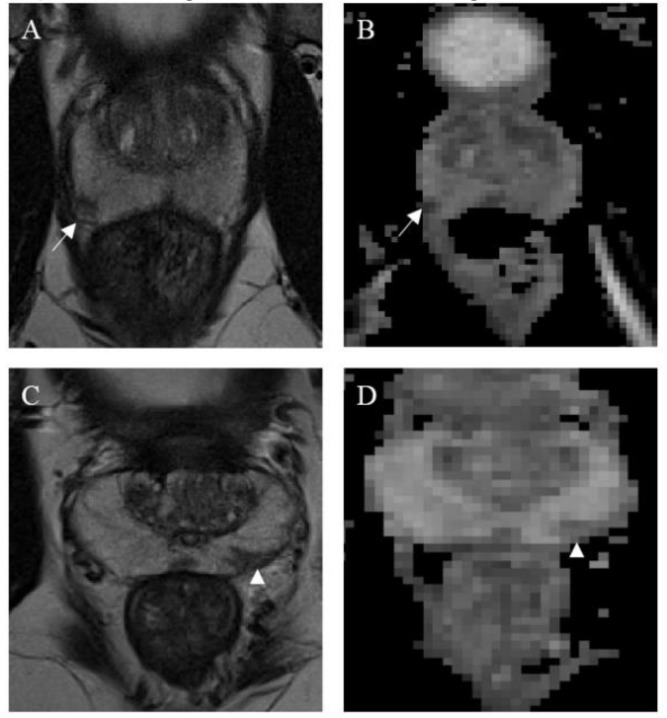
Magnetic resonance images from a clinically significant (**A**,**B**) and a non-clinically significant (**C**,**D**) prostate cancer lesion. Both are located in the peripheral zone; the clinically significant lesion, found in the right middle-posterior location (white arrows), appears as a nodular and well-defined area of low signal on both T2w (**A**) and ADC map (**B**). Conversely, the non-clinically significant lesion, found in the left middle-posterior location (white arrow heads), appears wedge-shaped, with no bulging on T2w (**C**) and only is mildly hypointense on the ADC map (**D**). Both lesions were deemed worthy of targeted biopsy.

A total of 299 verified prostate lesions were included in this study. Specifically, the lesion annotation masks were obtained from an online open repository (https://github.com/rcuocolo/PROSTATEx_masks, accessed on 1 July 2020) and coupled with the source MRI images, which can be found in the PROSTATEx training dataset (https://wiki.cancerimagingarchive.net/display/Public/SPIE-AAPM-NCI+PROSTATEx+Challenges, accessed on 1 July 2020) [16,17]. The ground-truth of the public dataset is obtained with a manual annotation. The 3 × 3 lesion and gland zone coordinate masks, freely available on a public repository (https://github.com/rcuocolo/PROSTATEx_masks, accessed on 1 July 2020), were retrieved by slice-by-slice seg-mentation on T2-weighted (T2w) and apparent diffusion coefficient (ADC) images, by the residents, with a subsequent check and eventual refinement by a radiologist. Of these 299 prostate lesions, 76 harbored clinically significant prostate cancer (cut-off = Gleason grade group ≥ 2) [18]. T2w and ADC maps images were used for the extraction of radiomic features. Images were obtained by two Siemens 3T MRI scanners, the MAGNETOM Trio and Skyra, without an endorectal coil. The acquisition of T2-w images was performed using a turbo-spin echo sequence with a resolution of around 0.5 mm in plane and a slice thickness of 3.6 mm. The ADC map was acquired by the scanner software from the diffusion-weighted imaging (DWI) (a single-shot echo planar imaging sequence with a resolution of 2 mm in-plane and 3.6 mm slice thickness, and with diffusion-encoding gradients in three directions) with three b-values (50, 400, and 800). Several algorithms were used to standardize signal intensity. Specifically, the T2-estimate map was obtained by using the MRI signal equation with an automated process [19] and the ADC map was automatically acquired from the diffusion-weighted images using the MRI scanner software. Figure 2 shows a clinically significant and a clinically non-significant lesion.

### 2.2. Radiomics Features Extraction

Images underwent a preprocessing stage before feature extraction, including resampling to isotropic voxel, the normalization of pixel intensity values and discretization [20]. A freely accessible software (PyRadiomics, v 3.0) was used for image pre-processing and feature extraction [21]. Z-score normalization was paired with scaling by a factor of 100 and a grey level value shift of +300, resulting in a final expected intensity range of 0–600. Discretization prior to first-order feature extraction was implemented using a fixed bin width of 5. Laplacian Gaussian filtering (sigma values= 1, 2, 3, 4, 5) and wavelet decomposition (all high- and low-pass filter combinations along the three axes) were applied, in addition to the original images. These settings were based on recommendations from the software developers and previous experiences in the literature [22]. Feature stability was tested for multiple segmentations on a random sample of 30 lesions (in total, masks from three operators were used), by calculating intraclass correlation coefficient and using a cut-off of 0.75. Low variance features were then excluded using a variance threshold of 0.01. Highly intercorrelated features (Pearson pairwise correlation > 0.8) were discarded, leaving a final number of 60 stable, informative features. Radiomic features are subsequently extracted to the prostate segmentation to simplify the detection, similarly to a previous published study [23]. A detailed description of the extracted radiomic features is available in the official PyRadiomics documentation (https://pyradiomics.readthedocs.io/en/latest/features.html, accessed on 1 July 2020).

### 2.3. Statistical Analysis

An inferential statistical analysis was performed by means of Levene’s test to assess the equality of variances for each feature of the two classes. Moreover, an unpaired *t*-test was carried out to assess the differences in the mean values for each feature between the two classes. Both statistic tests were implemented assuming a two-tailed distribution and a confidence level equal to 95% (definition of statistical significance: *p*-value < 0.05). The main purpose of this analysis was to understand whether the radiomics features extracted from the images could distinguish the significance of the lesion.

SPSS Software for Statistics v. 25 was used to perform the statistical analysis.

### 2.4. Machine Learning

Afterward, a ML analysis was conducted to evaluate the predictive power of the extracted features in classifying significant and non-significant lesions.

The following ML algorithms were implemented.

Decision Tree (DT) is based on an ordinary tree structure, which is made-up of a root, nodes, branches and leaves [24]. A DT starts from the root, then moves downward. The node from which the tree starts is named the root node, while the node where the chain ends is named the leaf node. Two or more branches can be extended from each internal node; in this case, it is not a leaf node. A node represents a certain feature while the branches represent a range of values [8]. J48 DT, which uses the C4.5 algorithm [25], was considered in the present work.

Random Forest (RF) [26], considered a classification task, is an ensemble of unpruned classification trees generated from the random selection of training set instances. Random features are selected in the induction process. A prediction is made by aggregating the ensemble predictions using the majority vote strategy. The Information Gain Ratio was used as a split criterion.

Gradient Boosted Tree (GBT) builds one DT at a time to fit the residual of the trees that precede it [27]. In the case of a binary classification, as in this study, a scalar score function is formed to distinguish the two classes. Given the training data and the classes related to each training instances, the goal of GBT is to choose a classification function that minimizes the aggregation of some specified loss function [27].

Ada Boost (ADA-B) is part of the boosting algorithms, in which several individual classifiers, DT in the case under study, are produced iteratively, and each classifier tries to accurately classify the training data [28]. The classifier uses an adaptive resampling strategy to choose the training samples. Each iteration assigns a weight to the dataset so that the next integration concentrates on reweighted datasets that were previously misclassified. The final classifier is a weighted sum of the ensemble predictions [29]. The advantage of the ADA-B algorithm is significant for solving several issues, including two-class problems, as in the case under study.

Naïve Bayes (NB) is based on the assumption that features are independent within a class in order to simplify the learning process [30]. Although this is an unrealistic assumption, NB competes well with more sophisticated classifiers [31], finding concrete applications in several scenarios including medical diagnosis [32].

K Nearest Neighbor (KNN) requires, in addition to training data, a fixed k value to search the k-nearest data based on distance computation. If the k found instances of different class labels, the classifier predicts that the class of the unknown example would be the same as the majority class [33]. Different distance metrics have been proposed in the scientific literature; for our purpose, we considered the Euclidian distance.

Two workflows of analyses were carried out using two different validation strategies for all the ML algorithms.

The first analysis used a 10-fold cross-validation to validate the predictive models by including all 60 radiomics features [34].

The second analysis used a hold-out validation; the dataset was divided into two non-overlapping parts and these two parts were used for training (70%) and testing (30%), respectively. This validation allows to avoid the problem of overfitting that is present in a re-substitution validation to be removed [35]. This analysis was performed using a feature selection method by means of a wrapper method based on backward feature elimination [36]. The usefulness of this method relies on the elimination of useless features and the building of a more reliable model based on a reduced set of features.

The main difference between the models was the presence of a feature selection step.

The performance of the proposed predictive models was evaluated through the following evaluation metrics: accuracy, sensitivity, specificity, area under the receiver operating characteristic curve (AUC-ROC) [37] and accuracy max, computed as the maximum value among the accuracies obtained in the ten cycles of 10-fold cross-validation.

ML algorithms were implemented through the artificial intelligence platform Knime Analytics Platform (version 3.7.1), which is increasingly diffused in the scientific literature [38,39,40] and has achieved an interesting performance when compared with other platforms and programming languages.

## 3. Results

The following subsections show the univariate statistical analysis results for the radiomics features and the ML analyses.

Altogether, 466 out of the 2576 features were considered stable after the inter-observer intra-class correlation analysis. An additional reduction was performed by removing zero variant features (n = 54 removed). Then, 352 out of the remaining 412 were excluded due to their high pairwise correlation, leaving 60 radiomic parameters in the dataset.

### 3.1. Statistical Analysis

Levene’s test was employed to verify the equality of variances, and then the univariate statistical analysis was performed through a *t*-test. Table 1 shows the descriptive statistics and the *p*-value of the *t*-test for all the radiomic features.

The statistical analysis showed that 16 features, out of a total of 60, were useful to distinguish a significant from a non-significant lesion (*p*-value < 0.05).

### 3.2. Machine Learning Analysis

The ML analysis was performed twice.

First, all 60 radiomics features were given as input to the six algorithms and the 10-fold cross-validation was employed to compute the evaluation metrics; the results of this analysis are shown in Table 2.

From this, the following results can be seen: the best algorithms were RF, according to their accuracy (77.9%), NB, which achieved the highest sensitivity (56.6%), KNN, with the highest specificity (91.9%), and ADA-B, which obtained the best AUCROC (0.720) and the highest accuracy max (86.7%).

Then, the dataset was divided, with 70% in the training set, and 30% in the test set, as per hold-out cross-validation. The training set was used to perform backward feature elimination, starting from all 60 features, and a set of variables was chosen for each algorithm. Finally, the evaluation metrics were computed on the test set for each algorithm by implementing 10-fold cross-validation. The results are shown in Table 3.

From this, the following results can be seen: the best algorithms were RF, again according to accuracy (82.1%) and also regarding specificity (91.0%), J48, which achieved the highest sensitivity (56.5%), and GBT, which obtained the best AUCROC (0.774). GBT and J48 achieved the highest accuracy max during the 10-fold cross-validation (100%). The application of backward feature elimination on the best algorithm, RF, made the algorithm select 39 features, which are shown in the Appendix A.

## 4. Discussion and Conclusions

The present study describes 60 stable, uncorrelated and non-invariant radiomics features, extracted from MRI images, which previously underwent a quality assessment [16], and used to distinguish significant from non-significant prostate cancer lesions through an ML approach. Firstly, a univariate statistical analysis was performed to prove that these 60 features were useful in distinguishing the lesions by themselves (16 of them were revealed to be statistically significant). Secondly, J48, ADA-B, RF, GBT, NB and KNN were implemented twice: i) they were applied with a 10-fold cross-validation on all 60 features; ii) a different ML workflow was employed, including a backward feature elimination strategy to identify the best subset of features, maximizing the evaluation metrics (i.e., accuracy).

Several studies have used a similar approach, combining radiomics and ML, for the diagnosis and characterization of prostatic lesions, aiming to differentiate clinically significant from non-significant lesions, and thus to stratify patient’s risk [13]. This differentiation is considered crucial in the management of prostate cancer patients for different causes: i) a growing number of prostate lesions, discovered through prostate-specific antigen (PSA) screening, are often clinically insignificant [41]; ii) in cases of clinically non-significant prostate cancer, the method of choice is active surveillance, whereas clinically significant lesions undergo surgical and medical treatment [42]; iii) then, the definition of clinically significant cancer becomes even more urgent [43].

A further analysis by subgroups showed that eight groups had used an ML approach while four used deep learning [44,45,46,47]. In the latter case, the used algorithms were convolutional neural network, artificial neural network and transfer deep learning with a pooled AUC-ROC of 0.78. Instead, in the former case, the ML algorithms used were NB, linear regression, RF, logistic regression, and support vector machine, with a pooled AUC-ROC of 0.90. 

Another interesting finding is the variability of the sequences used; three studies [48,49,50] employed a similar approach to ours, relying on T2 and ADC acquisitions with a pooled AUC-ROC of 0.90. Abraham et al. [44] and Bonekamp et al [51] also associated DWI with a pooled AUC-ROC of 0.81, which presented a lower stability in the extracted radiomic features [52]. Similarly, the use of automated analysis on T1- and T2-w sequences—without the need for gadolinium-based contrast medium—was also recently described [53,54].

Dynamic contrast-enhanced sequences were combined with baseline T2 and ADC sequences in two studies [55,56], resulting in a pooled AUC-ROC of 0.85. In addition, other studies extracted radiomic features from images of advanced MRI sequences that are not normally used in prostate MRI protocols, limiting the resulting algorithm’s clinical applicability [16].

Moreover, among the studies analyzed by Cuocolo et al., only five, like ours, started from a public archive, the Cancer Imaging Archive (https://www.cancerimagingarchive.net/, accessed on 1 July 2020) [44,45,46,47,48,57]. The others were based on data from single institutions, thus limiting the reproducibility and standardization of the algorithms used.

Of note, Papa et al. proposed a deep neural network architecture for classifying clinically significant prostate lesions of non-contrast-enhanced MRI images using Conditional Random Fields as a Recurrent Neural Network to enhance the classification performance [58]; although high evaluation metrics were achieved in this research, the proposed scores were affected by a high level of variability.

However, the present investigation evaluated a public dataset for improving the consistency of our technique, whereas most of the published studies are based on data from a single institution [13].

In addition, combining ML algorithms and radiomics has several advantages and potentialities. Since conventional image interpretation is based on radiologists’ experience, this technique could decrease inter-individual variability, as well as reporting time, leading to a potential benefit for less-experienced radiologists [59].

Moreover, the present paper demonstrated the usefulness of a ML and radiomics approach to images, which presents advantages, e.g., the non-necessity of a contrast medium. Therefore, the MRI acquisition protocol could be faster (by selecting only the most useful sequences) and cheaper, limiting the risk of possible side effects [60]. Indeed, the absence of a gadolinium-based contrast medium does not expose patients to different types of toxicities, such as nephrogenic systemic fibrosis, gadolinium brain accumulation and the invasiveness of intravenous access [61,62]. Moreover, an easier and faster protocol could also be more reproducible, allowing a better quality of images to be acquired in both local databases and public archives, in turn facilitating and implementing radiomic feature extraction and ML application. The present study has some limitations. Firstly, we did not demonstrate a potential association between the Gleason grading with clinical outcomes. Secondly, the performance of the used technique needs to be confirmed with further investigations. Thirdly, we cannot consider the histopathological variants.

In conclusion, the ML and radiomics approach, based on a public dataset, demonstrated a successfully discriminating, clinically significant prostate cancer. In the future, this radiomic signature could be interpreted as a “virtual biopsy”, which could potentially help to reduce the number of invasive procedures that are currently performed, and also guide the management of patients.

## Figures and Tables

**Table 1:** Descriptive statistics for the 60 radiomic variables and comparison between significant (0) and non-significant (1) lesions. Feature names are structured as follows: “MRI sequence”, “image type (original or filtered)”, “feature class”, “feature name”, each separated by an underscore.

Variable	Class	Mean	±	std. dev.	*t*-Test *p*-Value
t2_original_shape_MeshVolume	0	0.11	±	0.13	0.003 **
	1	0.18	±	0.19	
t2_original_firstorder_10Percentile	0	0.47	±	0.20	0.002 **
	1	0.40	±	0.15	
t2_original_glcm_Imc2	0	0.65	±	0.26	0.007 **
	1	0.56	±	0.26	
t2_original_glcm_Idm	0	0.59	±	0.16	0.098
	1	0.62	±	0.15	
t2_logsigma10mm3D_firstorder_90Percentile	0	0.24	±	0.15	0.119
	1	0.21	±	0.13	
t2_logsigma10mm3D_ngtdm_Busyness	0	0.15	±	0.15	0.042 *
	1	0.20	±	0.19	
t2_logsigma10mm3D_gldm_DependenceVariance	0	0.37	±	0.21	0.001 ***
	1	0.46	±	0.21	
t2_logsigma20mm3D_firstorder_90Percentile	0	0.44	±	0.19	0.874
	1	0.44	±	0.15	
t2_logsigma20mm3D_glcm_DifferenceVariance	0	0.19	±	0.16	0.506
	1	0.17	±	0.15	
t2_logsigma20mm3D_glszm_LargeAreaLowGrayLevelEmphasis	0	4.76	±	1.17	0.345
	1	6.16	±	9.36	
t2_logsigma30mm3D_glcm_Contrast	0	0.15	±	0.13	0.112
	1	0.12	±	0.11	
t2_logsigma30mm3D_glrlm_LongRunEmphasis	0	0.25	±	0.17	0.015 *
	1	0.31	±	0.19	
t2_logsigma30mm3D_ngtdm_Busyness	0	0.15	±	0.15	0.181
	1	0.17	±	0.13	
t2_logsigma40mm3D_firstorder_10Percentile	0	0.63	±	0.17	0.072
	1	0.66	±	0.12	
t2_logsigma40mm3D_firstorder_90Percentile	0	0.44	±	0.17	0.033 *
	1	0.49	±	0.14	
t2_logsigma40mm3D_firstorder_InterquartileRange	0	0.38	±	0.17	0.571
	1	0.39	±	0.19	
t2_logsigma40mm3D_glcm_Idm	0	0.50	±	0.17	0.054
	1	0.54	±	0.16	
t2_logsigma40mm3D_glcm_InverseVariance	0	0.85	±	0.11	0.325
	1	0.86	±	0.08	
t2_logsigma40mm3D_glszm_SizeZoneNonUniformity	0	0.07	±	0.10	0.064
	1	0.11	±	0.14	
t2_logsigma50mm3D_firstorder_Minimum	0	0.58	±	0.17	0.708
	1	0.57	±	0.14	
t2_logsigma50mm3D_firstorder_Variance	0	0.17	±	0.15	0.026 *
	1	0.22	±	0.17	
t2_logsigma50mm3D_glcm_Autocorrelation	0	0.16	±	0.15	0.006 **
	1	0.22	±	0.16	
t2_logsigma50mm3D_glcm_Contrast	0	0.11	±	0.11	0.465
	1	0.10	±	0.82	
t2_logsigma50mm3D_glrlm_LongRunEmphasis	0	0.26	±	0.16	0.413
	1	0.28	±	0.18	
t2_logsigma50mm3D_glszm_LargeAreaEmphasis	0	5.40	±	1.16	0.172
	1	7.50	±	1.13	
t2_logsigma50mm3D_gldm_LargeDependenceHighGrayLevelEmphasis	0	0.09	±	0.10	0.003 **
	1	0.13	±	0.11	
t2_waveletLLH_glcm_JointEnergy	0	0.28	±	0.19	0.927
	1	0.28	±	0.15	
t2_waveletLHL_firstorder_90Percentile	0	0.21	±	0.15	0.213
	1	0.19	±	0.12	
t2_waveletLHH_glcm_JointEnergy	0	0.45	±	0.21	0.305
	1	0.47	±	0.17	
t2_waveletHLL_glrlm_LongRunEmphasis	0	0.36	±	0.16	0.217
	1	0.38	±	0.17	
t2_waveletHLL_ngtdm_Busyness	0	0.14	±	0.14	0.059
	1	0.17	±	0.17	
t2_waveletHHL_firstorder_Variance	0	0.24	±	0.14	0.244
	1	0.22	±	0.11	
t2_waveletHHL_glszm_LargeAreaLowGrayLevelEmphasis	0	0.40	±	0.08	0.386
	1	0.06	±	0.15	
t2_waveletHHL_ngtdm_Busyness	0	0.17	±	0.17	0.047 *
	1	0.22	±	0.19	
t2_waveletLLL_firstorder_Energy	0	0.10	±	0.14	0.131
	1	0.13	±	0.15	
adc_original_firstorder_10Percentile	0	0.58	±	0.16	0.001 ***
	1	0.47	±	0.18	
adc_original_glrlm_LongRunEmphasis	0	0.31	±	0.18	0.612
	1	0.32	±	0.17	
adc_logsigma10mm3D_glcm_Contrast	0	0.10	±	0.11	0.718
	1	0.09	±	0.09	
adc_logsigma10mm3D_glcm_Idm	0	0.54	±	0.21	0.265
	1	0.57	±	0.19	
adc_logsigma10mm3D_ngtdm_Strength	0	0.11	±	0.15	0.070
	1	0.09	±	0.09	
adc_logsigma30mm3D_firstorder_90Percentile	0	0.11	±	0.14	0.001 ***
	1	0.09	±	0.09	
adc_logsigma30mm3D_glcm_DifferenceAverage	0	0.35	±	0.19	0.163
	1	0.38	±	0.16	
adc_logsigma30mm3D_glrlm_LongRunEmphasis	0	0.26	±	0.16	0.140
	1	0.23	±	0.12	
adc_logsigma30mm3D_glszm_GrayLevelNonUniformity	0	0.16	±	0.13	0.001 ***
	1	0.25	±	0.21	
adc_logsigma40mm3D_glcm_InverseVariance	0	0.66	±	0.16	0.694
	1	0.65	±	0.16	
adc_logsigma40mm3D_glszm_LargeAreaHighGrayLevelEmphasis	0	4.28 × 10^2^	±	1.22 × 10^2^	0.472
	1	5.39 × 10^2^	±	9.64 × 10^2^	
adc_logsigma50mm3D_firstorder_10Percentile	0	0.50	±	0.18	0.100
	1	0.54	±	0.19	
adc_logsigma50mm3D_glrlm_RunPercentage	0	0.58	±	0.15	0.051
	1	0.62	±	0.12	
adc_logsigma50mm3D_glszm_ZoneVariance	0	6.50 × 10^2^	±	1.39 × 10^1^	0.720
	1	5.99 × 10^2^	±	8.49 × 10^2^	
adc_waveletLLH_glcm_JointEnergy	0	0.27	±	0.16	0.572
	1	0.29	±	0.18	
adc_waveletLLH_glrlm_LongRunEmphasis	0	0.19	±	0.12	0.015 *
	1	0.24	±	0.16	
adc_waveletLHL_firstorder_90Percentile	0	0.36	±	0.14	0.926
	1	0.36	±	0.13	
adc_waveletLHL_firstorder_Kurtosis	0	0.10	±	0.10	0.004 **
	1	0.15	±	0.14	
adc_waveletHLL_firstorder_90Percentile	0	0.22	±	0.12	0.060
	1	0.25	±	0.12	
adc_waveletHLL_glcm_Imc2	0	0.46	±	0.23	0.984
	1	0.46	±	0.20	
adc_waveletHLL_glcm_Idm	0	0.53	±	0.16	0.743
	1	0.53	±	0.14	
adc_waveletHLL_glrlm_RunVariance	0	0.28	±	0.15	0.05 *
	1	0.32	±	0.18	
adc_waveletHHL_glcm_Contrast	0	0.07	±	0.12	0.834
	1	0.08	±	0.10	
adc_waveletHHL_glszm_LargeAreaEmphasis	0	4.70 × 10^2^	±	8.20 × 10^2^	0.064
	1	8.26 × 10^2^	±	1.58 × 10^1^	
adc_waveletLLL_glcm_Imc2	0	0.88	±	0.17	0.477
	1	0.89	±	0.12	

* = 0.01 < *p* < 0.05; ** = 0.001 < *p* < 0.01; *** = *p* < 0.001.

**Table 2:** Evaluation metrics (%) of the models computed on all 60 features with the 10-fold cross-validation.

Algorithms	Accuracy	Accuracy Max	Sensitivity	Specificity	AUCROC
J48	74.2	83.3	35.5	87.4	0.567
ADA-B	74.6	86.7	42.1	85.7	0.720
RF	77.9	83.3	48.7	87.9	0.713
GBT	74.9	86.2	34.2	88.8	0.682
NB	68.9	80.0	56.6	73.1	0.650
KNN	73.2	76.7	18.4	91.9	0.643

**Table 3:** Evaluation metrics (%) of the models computed using hold-out, backward feature elimination, 10-fold cross validation.

Algorithms	Number of Features	Accuracy	Accuracy Max	Sensitivity	Specificity	AUCROC
J48	16	82.2	100	56.5	91.0	0.635
ADA-B	14	81.1	88.9	52.2	91.0	0.708
RF	39	82.2	88.9	39.1	97.0	0.730
GBT	50	76.7	100	43.5	88.1	0.774
NB	15	70.0	88.9	21.7	86.6	0.546
KNN	25	74.4	88.9	30.4	89.6	0.676

## Data Availability

Data for this study can be found at https://github.com/rcuocolo/PROSTATEx_masks and https://wiki.cancerimagingarchive.net/display/Public/SPIE-AAPM-NCI+PROSTATEx+Challenges (accessed on 1 July 2020).

## Appendix A

Features selected by Random Forests through backward feature elimination:

1. t2_log-sigma-3-0-mm-3D_glcm_Contrast
2. t2_log-sigma-3-0-mm-3D_ngtdm_Busyness
3. t2_log-sigma-4-0-mm-3D_firstorder_10Percentile
4. t2_log-sigma-4-0-mm-3D_firstorder_90Percentile
5. t2_log-sigma-4-0-mm-3D_firstorder_InterquartileRange
6. t2_log-sigma-4-0-mm-3D_glcm_Idm
7. t2_log-sigma-4-0-mm-3D_glcm_InverseVariance
8. t2_log-sigma-5-0-mm-3D_firstorder_Minimum
9. t2_log-sigma-5-0-mm-3D_glcm_Contrast
10. t2_log-sigma-5-0-mm-3D_glszm_LargeAreaEmphasis
11. t2_log-sigma-5-0-mm-3D_gldm_LargeDependenceHighGrayLevelEmphasis
12. t2_wavelet-LLH_glcm_JointEnergy
13. t2_wavelet-LHL_firstorder_90Percentile
14. t2_wavelet-LHH_glcm_JointEnergy
15. t2_wavelet-HLL_glrlm_LongRunEmphasis
16. t2_wavelet-HHL_firstorder_Variance
17. t2_wavelet-HHL_glszm_LargeAreaLowGrayLevelEmphasis
18. t2_wavelet-HHL_ngtdm_Busyness
19. t2_wavelet-LLL_firstorder_Energy
20. adc_original_firstorder_10Percentile
21. adc_original_glrlm_LongRunEmphasis
22. adc_original_glszm_LargeAreaEmphasis
23. adc_log-sigma-1-0-mm-3D_glcm_Contrast
24. adc_log-sigma-1-0-mm-3D_glcm_Idm
25. adc_log-sigma-3-0-mm-3D_firstorder_90Percentile
26. adc_log-sigma-3-0-mm-3D_glrlm_LongRunEmphasis
27. adc_log-sigma-3-0-mm-3D_glszm_GrayLevelNonUniformity
28. adc_log-sigma-4-0-mm-3D_glcm_InverseVariance
29. adc_log-sigma-4-0-mm-3D_glszm_LargeAreaHighGrayLevelEmphasis
30. adc_log-sigma-5-0-mm-3D_glrlm_RunPercentage
31. adc_log-sigma-5-0-mm-3D_glszm_ZoneVariance
32. adc_wavelet-LHL_firstorder_Kurtosis
33. adc_wavelet-HLL_firstorder_90Percentile
34. adc_wavelet-HLL_glcm_Imc2
35. adc_wavelet-HLL_glcm_Idm
36. adc_wavelet-HLL_glrlm_RunVariance
37. adc_wavelet-HHL_glcm_Contrast
38. adc_wavelet-HHL_glszm_LargeAreaEmphasis
39. adc_wavelet-LLL_glcm_Imc2
